# Efficiency of IL-6 in Early Prognosis and Follow-Up in Critically Ill Patients with Septic Shock

**DOI:** 10.3390/diseases12110298

**Published:** 2024-11-20

**Authors:** Yenifer Gamarra-Morales, Jorge Molina-López, Felipe-Carlos Santiago-Ruiz, Lourdes Herrera-Quintana, Héctor Vázquez-Lorente, Félix Gascón-Luna, Elena Planells

**Affiliations:** 1 Clinical Analysis Unit, Santa Ana Hospital, 18600 Motril, Spain; 2 Faculty of Education, Psychology and Sports Sciences, University of Huelva, 21007 Huelva, Spain; 3 Intensive Care Unit, Santa Ana Hospital, 18600 Motril, Spain; felipecsr86@gmail.com; 4 Department of Physiology, School of Pharmacy, Institute of Nutrition and Food Technology “José Mataix”, University of Granada, 18071 Granada, Spain; lourdesherrera@ugr.es (L.H.-Q.); hectorvazquez@ugr.es (H.V.-L.); elenamp@ugr.es (E.P.); 5 Clinical Analysis Unit, Valle de los Pedroches Hospital, 14400 Córdoba, Spain; felix.gascon.sspa@juntadeandalucia.es

**Keywords:** interleukin-6, inflammation, septic shock, mortality, critical care patient

## Abstract

**Background/Objectives:** The aim of this study was to investigate the response of interleukin-6 (IL-6) during the first few hours of a patient’s stay in the Intensive Care Unit (ICU) in a sample of critically ill patients with septic shock, compared to healthy subjects as controls. Additionally, the study examined the association of IL-6 with morbidity and mortality in these patients, as well as its relationship with biomarkers such as lactic acid, C-reactive protein (CRP) and procalcitonin (PCT). **Methods:** This was a prospective analytical study involving 28 critically ill patients with septic shock, monitored from ICU admission through to their first three days of stay. Demographic data, comorbidities and clinical information, including IL-6 and severity scores, were recorded. **Results:** IL-6 levels were significantly higher in patients with septic shock compared to healthy subjects (*p* < 0.001) upon admission. IL-6 levels decreased by the third day of ICU stay (*p* < 0.005). An association between IL-6 and mortality was observed (areas under the curve 0.826, confidence interval (CI) 95% 0.659−0.994, *p* < 0.008). Significant correlations between IL-6 and lactic acid (*p* < 0.009 and *p* < 0.018) and partial thromboplastin time (*p* < 0.004 and *p* < 0.007) were found on the first and third days, respectively. IL-6 was also the correlated with an anion gap at admission to the ICU (*p* < 0.009). **Conclusions:** In conclusion, this study suggests that IL-6 could be a valuable marker for early sepsis follow-up in ICU patients, particularly during the first 72 h of hospitalization, providing important prognostic information in patients with septic shock.

## 1. Introduction

Septic shock is a disease with high mortality and morbidity that leads to a high consumption of resources in the Intensive Care Unit (ICU) [1,2]. In patients with this disease, there are severe circulatory, cellular and metabolic alterations that increase the risk of mortality, with mortality due to sepsis alone reaching over 40% [1].

Inflammation biomarkers are useful in the diagnosis of infections in emergency areas [3]. One of the difficulties that can be found in these emergency areas is differentiating sepsis from a non-infectious inflammatory response syndrome. A key biomarker studied in inflammatory diseases is interleukin-6 (IL-6), which is a glycoprotein involved in inflammation, whose release is induced by interleukin-1. It is secreted by macrophages, T cells, endothelial cells and fibroblasts. Elevated IL-6 levels have been associated with cardiovascular and all-cause mortality in the general elderly population [4]. IL-6 levels are also increased in a range of conditions involving inflammatory processes, including sepsis, neoplasms, autoimmune diseases, acquired immune deficiency syndrome (AIDS), alcoholic liver disease and infections or transplant rejection [5,6,7,8,9].

In sepsis, IL-6 peaks around two hours after infectious stimulation [10] and remains persistently elevated, often exceeding 500 pg/mL [11,12]. Notably, IL-6 levels rise earlier than those of procalcitonin (PCT) and C-reactive protein (CRP) and before the onset of fever [13,14,15]. IL-6 and PCT have a similar, yet superior, diagnostic value for sepsis compared to CRP [11,16]. IL-6, as an early marker in sepsis, may assist in diagnosing and predicting septic shock outcomes, providing valuable information alongside other parameters commonly used in clinical practice.

The primary objective of this study is to investigate the evolution of IL-6 levels over the first three days of ICU stay in critically ill patients with septic shock, and to compare these levels with those of healthy controls. The specific objectives include determining which marker—IL-6, CRP, PCT or lactic acid—is most effective for predicting the prognosis of sepsis. Additionally, the study aims to examine the association between IL-6 and 28-day morbidity and mortality, comparing IL-6 with other inflammatory markers to identify the best predictor of morbidity and mortality. Furthermore, the study explores the relationship between IL-6 and clinical parameters such as lactic acid, CRP and PCT in these patients.

## 2. Materials and Methods

This prospective analytical study examined the IL-6 levels of patients critically ill patients with septic shock on days 1 and 3 of their stay in the ICU. This is a pilot study. Over a two-year period (September 2015 to May 2017), adult patients (≥18 years old) admitted to ICU department were systematically screened for inclusion in this study. Patient selection followed the established protocols of the ICU at Virgen de las Nieves Hospital, Granada, Spain. The diagnosis of septic shock was based on the consensus criteria [17]. The sample size of this study was comparable to those reported in previous research [18,19]. Patients over 18 years of age with sepsis and severe arterial hypotension who were unresponsive to fluid therapy were included. Control samples were obtained from healthy adult subjects with a similar age to the study participants and blood values within the reference ranges.

The clinical and analytical parameters of these patients were collected on day 1 and day 3 of inclusion in the ICU. Clinical parameters included Acute Physiology and Chronic Health Evaluation II (APACHE II) score, sequential organ failure assessment (SOFA) score, days of mechanical ventilation, length of ICU stay and 28-day mortality. This study was approved by the Ethics Committee of the University of Granada (Ref.: 248/CEIH/2015). Patients who participated in the study did so after signing an informed consent. This study was conducted according to the principles of the Declaration of Helsinki, also in accordance with the International Conference on Harmonization/Good Clinical Practice Standards.

### 2.1. Biochemical Assessment

Fasting blood samples were drawn from ICU patients by venepuncture after the hemodynamic stabilization phase of admission and after 3 days of being in the ICU to measure renal function (ions and creatinine), liver function (bilirubin), nutritional parameters (folic acid, B12 and copper), hematimetric (hemoglobin, leukocytes, platelets, INR and activated partial thromboplastin time (APTT)) and inflammatory parameters (lactic acid, fibrinogen, lactate dehydrogenase (LDH)). PCR and PCT were performed by the hospital laboratory using standard techniques.

### 2.2. Assessment of Interleukin-6

The patients had blood drawn on day 1 and day 3 of their stay in the ICU. The samples were processed immediately; they were centrifuged at 3500 rpm for 10 min at 4 °C and frozen at −80 °C. IL-6 was measured in an Advia CentaurXP autoanalyzer from Siemens (Forchheim, Alemania), using competitive electrochemiluminescence immunoassays. The method used for the detection of IL-6 consists of a sandwich immunoassay [20]. In this method, the sample with IL-6 binds to anti-IL-6 mouse monoclonal antibodies labeled with acridinium ester. Paramagnetic particles coated with anti-IL-6 mouse monoclonal antibodies are also added to the reaction vessel. The IL-6 concentration of the sample is directly proportional to the amount of light measured by a luminometer.

### 2.3. Statistical Analysis

Qualitative variables were presented as frequencies and percentages, while quantitative variables were presented as arithmetic mean ± standard deviation (SD). The assumption of normality was tested using the Shapiro–Wilk test. The association of quantitative variables and mortality, as well as between case and control groups, was assessed using the U Mann–Whitney test. To verify whether IL-6 levels predicted mortality at baseline and during follow-up, the area under the curve (AUC) was calculated using the receiver operating curve (ROC) analysis, with comparison to healthy subjects. Correlations between quantitative variables were examined using Spearman’s correlation coefficient (Rho). The evolution of critically patients with septic shock during their stay in the ICU was analyzed by comparing quantitative variables between days 1 and 3 using the Wilcoxon test. Statistical significance was set at *p* < 0.05. The statistical analysis was performed using SPSS version 21.0 (IBM SPSS, Armonk, New York, NY, USA). GraphPad Prism version 9.0 software (GraphPad Software, San Diego, CA, USA) was used for plotting the graphs.

## 3. Results

When comparing IL-6 values in the group of healthy people with the group of patients with septic shock, a statistically significant difference was obtained between both groups (*p* < 0.001). It was observed that IL-6 increased in 100% of the patients admitted, while it remained very low (<6.4 pg/mL) in the healthy controls.

### 3.1. Patient Characteristics

Twenty-eight patients who were admitted to the ICU with septic shock were enrolled in this study. The demographic and clinical characteristics of the patients and the evolution of their condition after three days are shown in Table 1. The gender distribution of the sample was twenty-two male patients (78.6%) and six female patients (21.4%) with a mean age (SD) of 61.93 (14.12) years. Regarding the original cause of septic shock, 50% were due to an abdominal cause, 27% due to a respiratory cause and 23% were due to a urinary cause. The microorganisms that caused the infection were three Streptococci (10.7%), an Acinetobacter (3.6%), one Pseudomonas (3.6%), one Campylobacter (3.6%), one Clostridium (3.6%) and one Candida Albicans (3.6%), and the rest were E. coli (60.5%).

There was a significant decline in the SOFA on the third day (*p* < 0.011). A total of 15 patients needed mechanical ventilation (53.6%) and the mean length of stay in ICU was 7.04 (10.49) days. The observed 28-day mortality was 42.9% (12 patients). The control samples were taken from 28 healthy patients with normal test results and similar ages.

### 3.2. Biochemical Parameters

Table 2 shows the biochemical and hematological parameters of the critically ill patients and a comparative analysis between admission and the third day of ICU stay. The 28 cases of septic shock had highly abnormal laboratory parameters with very high levels of PCR and PCT (as acute markers of inflammation and infection). In the comparative analysis, there were statistically significant differences between the IL-6 values on the first and third days of evolution in the ICU (*p* < 0.005), decreasing on the third day. Moreover, there were statistically significant decreases in lactic acid, PCT, hemoglobin and platelets on the third day (*p* < 0.033). Finally, the IL-6 levels in the critically ill patients were compared with healthy controls, and statistically significant differences between the healthy controls and the patients with septic shock were observed (*p* < 0.001).

### 3.3. Interleukin-6 and Morbimortality Parameters

Table 3 describes the comparison of IL-6, platelets, and lactic acid between deceased and non-deceased critically ill patients on admission and on the third day in the ICU. After making a comparison, it was found that the IL-6 levels were different in the deceased patients compared to survivors, being higher in the deceased patients on both the first day (*p* < 0.008) and on the third day in the ICU (*p* < 0.016). There also were differences in lactic acid and platelets (*p* < 0.046), both on admission and the third day in ICU. 

**Figure 1:**
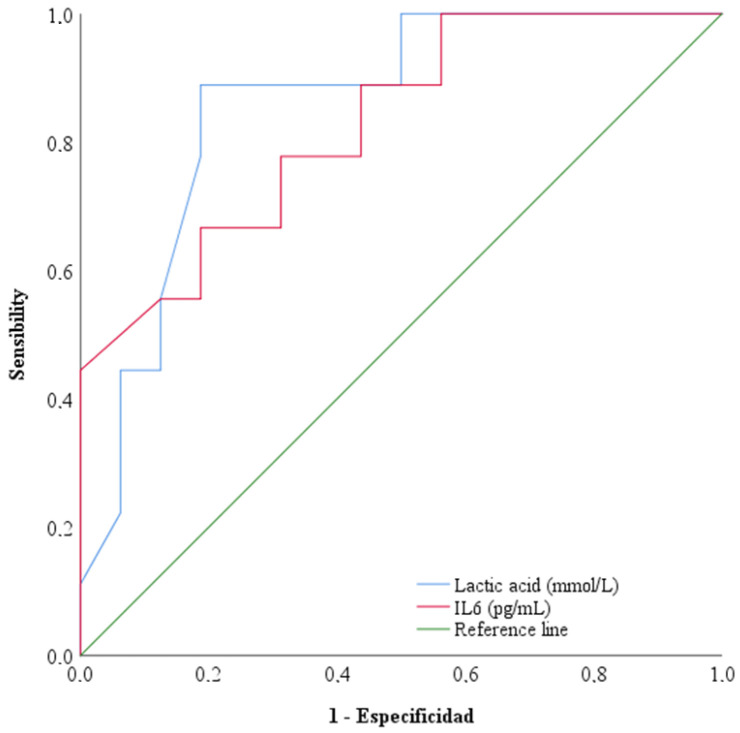
The receiver operating characteristic curves (ROCs) of IL-6 and lactic acid to check and compare the mortality prognostic value. The areas under the curve were represented for IL-6 and lactic acid on the first day in the ICU.

Figure 1 shows the receiver operating characteristic curves (ROCs) for IL-6 and lactic acid to check and compare their prognostic value for mortality. The areas under the curve were 0.826 (confidence interval (CI) 95% 0.659−0.994) for IL-6 on the first day and 0.858 (CI 95% 0.707−1.008) for lactic acid on the first day.

### 3.4. Interleukin-6, Acute Phase Reactants and Clinical Outcomes

**Figure 2:**
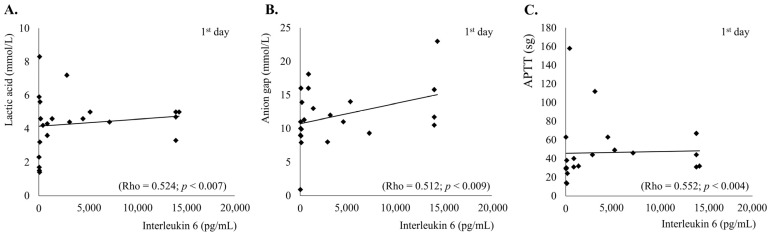
Correlations of interleukin-6 with other acute phase markers. (**A**) lactic acid vs. IL-6 correlation. (**B**) anion gap vs. IL-6 correlation. (**C**) APTT vs. IL-6 correlation.

There were statistically significant correlations between IL-6 and lactic acid, both on the first day (Rho = 0.524; *p* < 0.007) and on the third day (Rho = 0.641; *p* < 0.018); between IL-6 and GAP (Rho = 0.512; *p* < 0.009) on the first day; and between IL 6 and APTT on the first day (Rho = 0.552; *p* < 0.004) and on the third day (Rho = 0.706; *p* < 0.007) (Figure 2). With regard to clinical outcomes, no significant associations were observed with the severity of the SOFA and APACHE II scores and days of stay in the ICU.

## 4. Discussion

The main finding of this study was that IL-6 decreased on the third day following ICU admission in patients with septic shock. IL-6 was higher in patients with septic shock than in the control group of healthy people. Additionally, IL-6 was found to be a stronger predictor of mortality compared to the other parameters studied (PCT/CRP), as it demonstrated a larger area under the curve, similar to that of lactate. Another objective of the study revealed that IL-6 correlates with other sepsis markers, showing significant correlations with APTT, anion gap, and lactate.

In our study, the decrease in IL-6 levels on the third day of ICU stay could be due to the therapeutic measures implemented in the ICU for patient recovery. One study [21] suggests that IL-6 predicts treatment success in patients with non-surgical sepsis more effectively within first 48–72 h than PCT or CRP. These findings lead us to consider that, in addition to its role as a marker of inflammation or infection, IL-6 may also help to assess the efficacy of early interventional protocols in septic shock patients in the ICU.

The innate immune system can recognize microbial pathogens and endogenous alarmins [22], leading immune cells to release inflammatory mediators, which are responsible for the development of organ dysfunction. Upon ICU admission, it is important to distinguish critically ill patients with infectious causes from those with non-infectious causes. Measuring CRP, lactate, PCT and IL-6 in the clinical laboratory is essential for making a differential diagnosis and prognosis of the patient’s evolution. However, research that includes all four biomarkers in critically ill patients with organ dysfunction remains insufficient. In this study, we propose using this complete set of molecules to develop a comprehensive prognosis, ensuring that IL-6 is not excluded.

In the present study, IL-6 levels were much higher in the septic patients than in healthy subjects, as expected based on previous research [6]. It is well established that excessive plasma IL-6 plays a key role in the pathophysiology of septic shock, particularly since the administration of low doses of endotoxin to animals or human volunteers not only induces an increase in plasma TNF [23] but also an increase in IL-6 [24]. All of the cases in this study showed elevated IL-6 levels, with a 100% sensitivity for diagnosing sepsis, as reported in earlier studies [25,26]. Previous research has also highlighted the prognostic and diagnostic utility of IL-6 in pre-operative patients [27], septic patients [28,29] and COVID-19 patients [30]. Furthermore, studies have demonstrated a higher discriminatory power between severe sepsis and SIRS when using PCT and IL-6, with a reported area under the ROC of 0.923 [19]. In addition, IL-6 has been shown to be a better diagnostic marker for sepsis compared to PCT and CRP [25,31].

As for the relationship between the parameters and morbidity and mortality, our study shows a significant relationship with mortality, as patients who died had higher IL-6 levels. In a study very similar to ours [32], IL-6 was studied as a predictor of mortality after 72 h. Although no differences in IL-6 levels were found between septic patients who survived and those who did not, there was a significant increase in IL-6 at 72 h among patients who died. Other studies [6] have also reported similar results, showing an association between IL-6 and mortality in patients with sepsis, with a stronger association in those with septic shock. In another study [10], IL-6 was measured each day of admission in patients with sepsis, and no differences were observed between survivors and non-survivors on the first day of admission. However, higher IL-6 values were obtained in patients with septic shock than in patients with sepsis. In our study, all the patients had septic shock, which may explain the significant association of IL-6 with mortality on the third day of ICU admission. The discrepancy with previous studies, where this association was not observed, could be due to the inclusion of patients with sepsis but not septic shock [10,32].

SOFA is the gold standard for assessing the severity of sepsis in ICU patients; however, certain biomarkers have also been studied, as a whole, increasing the diagnostic performance and prognostic accuracy of predicting morbidity and mortality. For instance, both PCT and IL-6 were included in such analyses [26]. Previous studies have observed correlations between sepsis severity and serum levels of IL-6 [31] and PCT [33,34,35,36,37]. Moreover, IL-6 has been correlated with SOFA scores and low platelet levels [10], providing evidence of the usefulness of IL-6 in monitoring septic shock patients. On the other hand, lactic acid was also included in the definition of sepsis and septic shock in the 2016 consensus [1]. As in our study, lactic acid was previously described as a predictor of mortality in patients with infection in the emergency department [38]. In short, this biomarker has proven useful for the diagnosis, prognosis and evolution of the septic patient [1,38,39]. A correlation between IL-6 and lactic acid, as observed in our study, further supports the prognostic value of IL-6 [6]. Another study [40] showed that IL-6 is more strongly associated with all-cause mortality and cancer-related mortality, whereas CRP only primarily predicts cardiovascular mortality over a 16-year follow-up period. In addition, in the short term, IL-6 and CRP were more strongly associated with mortality than anion gap, as observed in our study. Altogether, these findings suggest that high IL-6 concentrations in septic shock patients are related to clinicopathological outcomes. We suggest IL-6 as an early follow-up marker, where its concentration decreases with the therapeutic measures applied in the ICU.

IL-6 levels have been included in a nutritional risk index in critical patients called NUTRIC, this scoring algorithm was proposed by Heyland et al. [41]. The NUTRIC was proposed to assess the risk of adverse events in critically ill patients. There are several articles that discuss the relationship between malnutrition in critically ill patients and adverse events in the ICU [42,43,44,45,46,47,48]. These adverse events may be modifiable through nutritional intervention.

Our study has a number of limitations that mean that the results must be interpreted with caution. Firstly, possible confounding factors in the subjects who participated in our study (sociodemographic and/or socioeconomic) were not evaluated and the subjects who participated came from the same hospital. For this reason, the results obtained cannot be extrapolated to other populations. Secondly, another sample of critically ill patients without septic shock could not be included to act as a control group. Thirdly, we must take into account that the alterations found in these subjects may be influenced by the heterogeneity of the patients, including their underlying conditions (diabetes mellitus, arterial hypertension, kidney disease, liver disease) or the severity of the disease (depending on the focus of the infection or the microorganism responsible for the infection). Further studies with a larger sample size are needed to be able to extrapolate these results to the rest of the population, as well as to obtain more definitive conclusions. This study could be repeated with a larger population of critically ill patients that includes patients without septic shock who can be used as a control group; this could help us validate the findings found in our study.

## 5. Conclusions

The present study shows that IL-6 could be useful as a marker in the follow-up of patients with sepsis and their response to intervention during the early days of their ICU stay. IL-6 could serve as a predictor of mortality in a similar way to lactic acid and as a predictor of morbidity, since an association with acute phase reactants is shown in this study.

## Figures and Tables

**Table 1:** Demographic and clinical characteristics of subjects and the evolution of condition after three days in critically ill septic shock patients with COVID-19.

	1ª Day Median (IQR)	3ª Day Median (IQR)	*p* Value
Age, (years)	52.0 (22.0)	-	*-*
Male, number (%)	22 (78.6)	-	*-*
SOFA score	12.0 (4.0)	9.0 (7.0)	**0.013**
APACHE II score	22.0 (11.0)	-	-
Sepsis focus, number (%)			
Respiratory (%)	14 (50.0)	-	-
Urinary (%)	6 (21.0)	-	-
Abdominal (%)	8 (29.0)	-	-
MAP (mmHg)	65.0 (18.0)	80.0 (43.0)	**0.012**
FiO_2_ (%)	0.60 (0.22)	0.40 (0.26)	**0.001**
PaO_2_/FiO_2_	232.0 (101.0)	242.0 (159.0)	0.583
PaCO_2_ (mmHg)	40.5 (20.0)	39.5 (14.5)	0.345
PEEP (cmH_2_O)	10.0 (5.5)	7.5 (4.5)	0.276
Cst (mL/cmH_2_O)	37.5 (15.25)	31.5 (12.25)	0.593

n = 28. Data are expressed as median (interquartile range) unless otherwise stated. The fourth column shows the statistical significance after applying Wilcoxon test for related samples; thus, the evolution is shown after three days. Significant *p*-values are represented in bold. *p*-values after applying the Benjamini–Hochberg (BH) procedure for controlling the false discovery rate (FDR) are presented. Statistical significance = *p* < 0.05. Abbreviations: MAP: mean arterial pressure. PaO_2_/FiO_2_: partial oxygen arterial pressure/fraction of inspired oxygen. PaCO_2_: partial pressure of carbon dioxide in arterial blood. PEEP: positive end expiratory pressure. Cst: static compliance.

**Table 2:** Biochemical parameters and the evolution of critically ill septic shock patients with COVID-19 after three days.

	Reference Values	1st Day Median (IQR)	3rd Day Median (IQR)	*p* Value 1st–3rd Day
Lactic acid (mmol/L)	0.6–2.5	4.35 (1.78)	1.55 (1.28)	**0.014**
Sodium (mmol/L)	136–146	137.0 (7.5)	138.5 (7.0)	0.589
Potassium (mmol/L)	3.5–5.1	3.80 (1.1)	3.80 (0.75)	0.850
Anion Gap (mmol/L)	7–16	11.50 (6.02)	9.50 (8.92)	0.079
Creatinine (mg/dL)	0.67–1.20	2.41 (3.09)	1.79 (2.94)	0.097
Total bilirubin (mg/dL)	0.3–1.2	1.04 (1.70)	1.40 (4.58)	0.975
Fibrinogen (mg/dL)	200–350	454.0 (210.0)	475 (219)	0.778
LDH (U/L)	110–295	645 (890)	577 (1814)	0.301
CRP (mg/L)	0.02–5	29.0 (15.4)	15.4 (5.5)	0.441
Procalcitonin (ng/mL)	<0.5	27.2 (12.9)	5 (18.63)	**0.018**
Leukocytes (*10^3^/µL)	3.5–10.5	11.69 (14.98)	13.24 (81.28)	0.679
Hemoglobin (g/dL)	11–17	10.70 (3.57)	8.75 (3.53)	**0.001**
Platelets (*10^3^/µL)	120–450	13.40 (13.03)	68.50 (86.75)	**0.033**
INR (ratio)	0.8–1.16	1.50 (0.33)	1.25 (0.26)	0.341
APTT (sg)	26–37	44.00 (41.50)	36.00 (14.00)	0.214
Interleukin-6 (pg/mL)	<4.4	2860.9 (10,531.2)	65.1 (335.2)	**0.005**

n = 28. Data are expressed as median (interquartile range). Fifth column shows the statistical significance after applying Wilcoxon test for related samples; thus, the evolution is shown after three days. Significant *p*-values are represented in bold. *p*-values after applying the Benjamini–Hochberg (BH) procedure for controlling false discovery rate (FDR) are presented. Statistical significance = *p* < 0.05. Abbreviations: LDH, lactate dehydrogenase; CRP, C-reactive protein.

**Table 3:** Association of some parameters with 28-day mortality in septic shock patients.

	1st Day (Mean ± SD)		3rd Day (Mean ± SD)		*p* Value 1st Deceased vs. Non-Deceased	*p* Value 3rd Deceased vs. Non-Deceased
Parameters	Non-Deceased	Deceased	Non-Deceased	Deceased		
Interleukin-6 (pg/mL)	2879.9 ± 4786.5	43,828.2 ± 71,858.4	83.7 ± 142.3	2238.6 ± 2560.3	0.007	**0.011**
Platelets (*10^3^/µL)	141.9 ± 88.5	96.2 ± 103.2	104.5 ± 55.6	29.3 ± 26.0	0.047	**0.015**
Lactic acid (mmol/L)	3.94 ± 1.77	5.76 ± 1.82	1.45 ± 0.36	5.22 ± 2.98	0.013	**0.001**

n = 28. Data are expressed as mean ± standard deviation. Mann–Whitney test for independent samples was used to compare between deceased vs. non-deceased groups of patients at 1st and 3rd day in ICU stay. Significant *p*-values are represented in bold.

## Data Availability

Data will be shared upon reasonable request by the corresponding authors: Yenifer Gamarra-Morales.
